# Ultrasonic-Aided Co-Precipitation of Tannins and Chitosan Ammonium Salt on Cotton Fabric for Antimicrobial and Ultraviolet-Shielding Properties: An Efficient, Colourless, and Eco-Finishing Strategy

**DOI:** 10.3390/ma15124367

**Published:** 2022-06-20

**Authors:** Yuyang Zhou, Feiyang Zheng, Jingjing Wang

**Affiliations:** 1 National Engineering Laboratory for Modern Silk, China National Textile and Apparel Council Key Laboratory of Natural Dyes, College of Textile and Clothing Engineering, Soochow University, Suzhou 215123, China; zhengfeiyang2022@163.com; 2 Key Laboratory of Clean Dyeing and Finishing Technology of Zhejiang Province, Shaoxing University, Shaoxing 312000, China; 3 Suzhou Quzhuanrang Information Technology Co., Ltd., Suzhou 215028, China

**Keywords:** bio-extract, cotton fibre, electrostatic co-deposition, ultrasonic technology, UV proof, antibacterial

## Abstract

Sustainable fabrication of protective cotton, using bio-extracts, is becoming increasingly attractive. However, many shortcomings—including the introduction of potentially hazardous mordants or modifiers to cotton, annoying colour changes after finishing, and low processing efficiency—require further melioration. Therefore, an efficient ultrasonic-assisted colourless finishing process was developed in this study, to fabricate ultraviolet-proof and antimicrobial cotton. A pair of oppositely charged bio-based substances, i.e., tannin acid (TA) and hydroxypropyltrimethyl ammonium chloride chitosan (HACC) were introduced during the ultrasonic process. The results reveal that cationic HACC significantly promotes the adsorption of TA to cotton. The apparent colour of the cotton remained almost unchanged after finishing. Based on *Pesudo first-*/*second-*order kinetic models, chemisorption was verified as the dominant mechanism. Efficiency under ultrasound was enhanced by 5.3% (70 °C) and 27% (90 °C), respectively. A mathematical modelling study established the factors to be in the following order of significance: concentration > pH > temperature. Under optimal conditions, a theoretical maximum UPF of 380.8 was achieved. TA (8 g/L)-treated cotton deactivated up to 98% of *Escherichia coli*, and also provided excellent UV-shielding performance. In general, the ultrasonic-assisted eco-dyeing and finishing process for cotton was explored in depth from practical and theoretical perspectives, which should push forward the development of the sustainable textile industry.

## 1. Introduction

Recently, there has been a growth in worldwide research into the integration of biodegradable fibres and functional reagents to mitigate environmental and health issues related to the release of micro-fibres and hazardous synthetic chemicals. Cotton made up over 80% of the total production of natural fibre in 2018 [1]. The global mission towards carbon neutrality is also driving conventional textile manufacturers to develop advanced and cleaner processes. The textile industry has been reported to account for approximately 10% of the world’s carbon emission, which is highly responsible for climate change. Thus, there is great demand for the development of an efficient treatment process for cotton, to mitigate this negative environmental impact, and to pursue the Sustainable Development Goals of the United Nations.

Cotton fabric suffers from high ultraviolet (UV) transmittance [2], and also provides a favourable environment for microbial growth [3], thus failing to protect the wearer, and even inducing skin diseases. A variety of synthetic UV absorbers and antimicrobials have been incorporated into cotton products; however, recent studies have confirmed their shortcomings, such as toxicity, poor activity, and weak washing fastness [4,5]. Furthermore, the corresponding process was not deemed to be ‘green’, due to its being potentially hazardous to the environment, and also due to its laying an additional financial burden on textile manufacturers, for wastewater treatment.

Ultrasonic-aided processing is a more efficient strategy than the conventional waterbath treatment [6,7]. Ultrasonic waves travel through the aqueous medium, and accelerate the movement of dyes or finishing reagents to fibres, thus saving on the total processing energy and time [8,9]. Fabric damage resulting from heating and long processing hours could also be prevented by an efficient ultrasonic-aided process. Therefore, there is great demand for the estimation of time-savings by ultrasound based on an in-depth adsorption mechanism, and to reveal the influence of ultrasound on the adsorption of reagents to textile substrates.

Renewable bio-materials are being incorporated, with ease, into fibres to achieve sustainable textiles [10,11]. It is more difficult for bio-based extracts to be directly applied to the natural fibres of cotton than to silk or wool, due to the lack of positive sites on cotton for anchoring bio-based extracts. At present, mordanting with metal ions, and cross-linking with additional reactive chemicals, are two common ways to address this issue [11,12,13,14,15]; however, these methods result in annoying colour changes and decreasing functionalities [11,16,17]. Although cotton fibres are often cationised using synthetic quaternary ammonium salts [18], the drawbacks—such as thermal instability, unpleasant odour, and potential health and environmental hazards—greatly hinder their application [18]. Therefore, the search for green and safe cationic alternatives for cotton is currently a popular research topic.

Chitins are one of the most popular agro-industrial by-products; they exist abundantly in the seafood industry, and their advantages include renewability, biocompatibility, biodegradability, and non-toxicity [19,20]. The deacetylation form of chitin is chitosan—a well-known, bio-based cationic polysaccharide [20], which possesses multiple functionalities, being antioxidant, antimicrobial, and anti-coagulant [21,22]. A variety of functions, including antimicrobial and flame-retardant properties, have been realised on fibres and polymers using chitosan [23,24]. However, the application of chitosan derivatives, to increase the affinity of natural molecules to cotton, requires further investigation. Tannin acid (TA) is another natural extract famous for its multiple biological activities, being antioxidant, antimicrobial, and anti-inflammatory. Many studies have treated silk and wool using TA, through an electrostatic depositing mechanism between the TA (δ−) and protein fibre (δ+). Nevertheless, such a process could not be directly transferred to cotton fibre, which has insufficient positively charged sites. Therefore, how to apply TA to cotton without heavily impacting the colour and functionality, requires further exploration.

**Figure 1:**
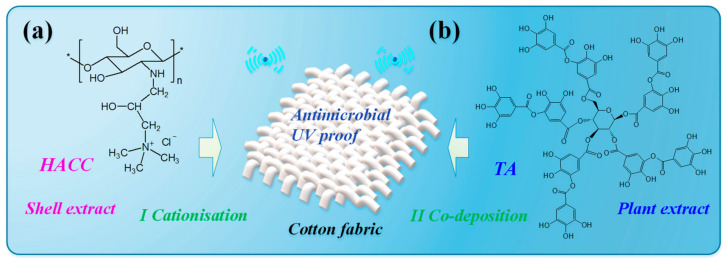
Ultrasonic-aided electrostatic co-deposition on cotton fabric using (**a**) HACC and (**b**) TA.

To solve the above issues, this study introduces an ultrasonic-aided strategy to fabricate fully bio-based antimicrobial and UV-proof cotton fabric via the electrostatic co-depositing of TA and chitosan derivative. Specifically, CTS derivatives were firstly applied to cotton to create positively charged binding sites (I Cationisation), followed by co-deposition with negatively charged TA (II Co-deposition) (Figure 1). This study aimed to simultaneously tackle two issues, i.e., (a) electro-repellence between cotton and anionic bio-extracts, and (b) the low adsorption efficiency of natural extract to textiles in conventional processes. Moreover, a chitosan derivative—hydroxypropyltrimethyl ammonium chloride chitosan (HACC) rather than chitosan—was introduced to the process, due to its better water-solubility, from the perspective of practical manipulation. The surface zeta potential, colour features, and antibacterial and UV-proof properties of modified cotton fabrics were evaluated and analysed.

## 2. Materials and Methods

### 2.1. Materials

Cotton fabric was bought from Henan Xinye Textile Co., Ltd., China. TA (Purity: 97%) was purchased from Sigma–Aldrich. HACC (Purity: 98%) was purchased from Xi’an Shouhe Biotechnology Co., Ltd., Xi’an, China. The other chemicals were of analytical grade. Deionized water was used.

### 2.2. Methods

#### 2.2.1. Cationisation

The cationisation of the cotton was carried out using HACC (4 g/L) in thermal controlling Ultrasonic Cleaners (Kunshan Ultrasonic Instrument Co., Ltd., Suzhou, China) integrated with a mechanical stirring system at 50 °C for 30 min. The resultant fabric was then cured at 120 °C for 2 min, and rinsed and dried for the following process.

#### 2.2.2. Adsorption Kinetics Study

**Figure 2:**
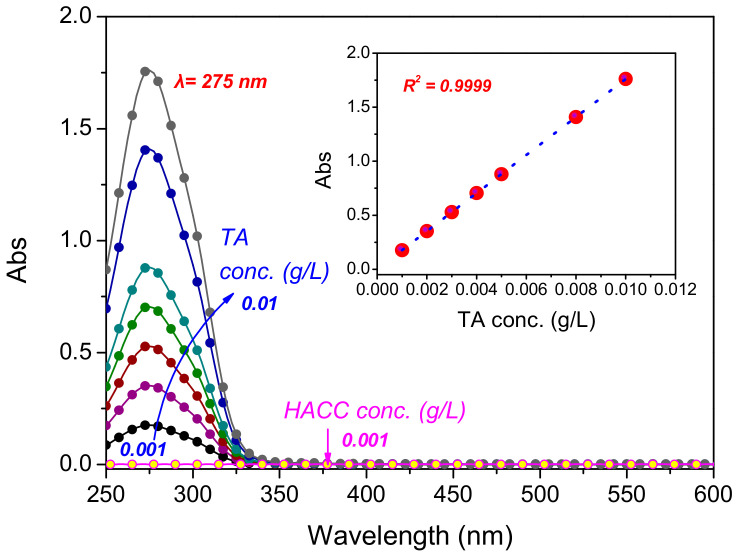
UV–Vis adsorption spectra of TA at various concentrations and HACC.

The adsorption of the TA onto the cationised cotton fabric was also processed, as described above, in the thermal controlling and mechanical mixing ultrasonic system at constant temperatures (70 and 90 °C). The pH was adjusted by citric acid-disodiumhydrogen phosphate buffer at 3.6 with a liquor ratio (mass of solution to fabric) of 50:1. The control fabric sample was prepared in a homothermal waterbath. The exhaustion rate was calculated according to the absorbance/concentration linearship at the λmax (275 nm), using Equation (1) (see Figure 2),

(1) $$E\%=\frac{W_{0}-{\;W}_{1}}{W_{0}}\;\times \;100$$

where *W*_0_ and *W*_1_ are the initial and final weights, respectively, of the TA in solution.

#### 2.2.3. CCD Experimental Design

A CCD experiment was carried out using Minitab 19 (USA, Trial edition). The time was set at 60 min, as this was close to the adsorption equilibrium known from the pre-trials. Three factors—TA concentration, pH, and temperature—indicating the UPF value of the cotton, were explored through CCD experiment. The variables and experimental design levels are shown in Table 1. The factorial matrix and the response values are displayed in Table 2. All processes were completed as per the directions given in Section 2.2.2, ‘Adsorption Kinetics Study’, above.

#### 2.2.4. Characterisation

*Colour features*: A computer colour measurement (SF600) and matching tool (Datacolor, Lawrenceville, NJ, USA) were adopted to determine the colour features, including *K*/*S* and L*a*b* values. Scanning electron microscope (SEM) images were taken on a Hitachi S-4800 SEM (Hitachi High-Technologies Co., Tokyo, Japan) at an acceleration voltage of 3.0 kV.

*Surface ζ-potential*: The fabric’s surface electric potential was determined on a SurPASS electrokinetic analyser (Anton Paar GmbH, Graz, Austria). A couple of pieces of squared fabric (10 × 20 mm^2^) were stabilized in the cell chamber, leaving a flowing channel in between. The current was measured using Ag/AgCl electrodes. The pH was changed in the range of 3–6 with HCl/NaOH.

*Functionalities*: The fabric’s antibacterial activity against *E. coli* was assessed using GB/T 20944.3-2008. Fabric pieces (0.75 g) were submerged with bacteria, and oscillated in a shaker at 30 °C for 24 h. The bacterium solution was then diluted 1 k times, injected on agar for cultivation, and kept at 37 °C for 24 h. Finally, the antibacterial activity was determined using Equation (2),

(2) $$\mathrm{Antibacterial rate}(\%)=\frac{A_{c}-A_{e}}{A_{c}}\;\times \;100$$

where *A_c_* and *A_e_* are the colony numbers from the control and experimental plates, respectively.

The UPF and UV transmittance were measured using a Labsphere UV transmittance meter (Labsphere Inc., Sutton, NH, USA). Each sample was examined four times in separate locations.

## 3. Results

### 3.1. UV–Vis Adsorption Study

A batch of adsorption curves of TA and HACC solutions in the UV and visible light regions are depicted in Figure 2. The intense peak of TA located at 275 nm is attributable to the π→π* transition in the aromatic ring of the TA structure [25,26], which was distinct from that of the HACC. There was a fine linear relationship between the solution absorbance and the concentration of the TA, with a strong correlation value (R^2^ = 0.9999), indicating good uniformity and stability. This feature was important in regard to the accurate measurement of the absorbance of the TA in solution, and the estimation of the TA adsorption quantity on the cotton fibres, as described in the following sections. It is notable that both the TA and the HACC had no adsorption in the visible light region, which indicated their colourless nature, and was consistent with previous studies [27,28]. This is one advantage of this research design, which aims to impart additional functions to cotton using bio-extracts, without obvious colour change.

### 3.2. Cationisation and Co-Deposition

**Figure 3:**
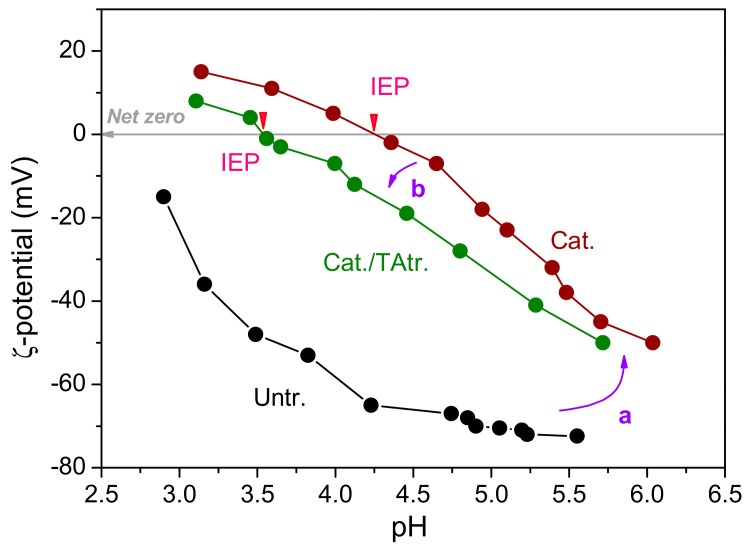
Surface ζ-potential change of Untr., Cat., and Cat./TAtr. cotton as a function of pH value. Note: Untr.: untreated; Cat.: cationised; Cat./TAtr.: cationised/TA-treated.

Figure 3 shows the ζ-potential of pure, cationised, and HACC/TA co-deposited cotton. The ζ-potentials of the untreated (Untr.) cotton were lower than zero, indicating it had almost no positive sites. Treated with HACC, the isoelectric point (IEP) of the cotton appeared at 4.2 (a), which demonstrated the increasing number of positive sites on the cotton. The IEP value of the treated cotton revealed a net zero charge rising from the integration of the hydroxyls of cotton and the trimethylammonium groups of HACC. The high ζ-potential of HACC-modified cotton attracted the negatively charged TA through electrostatic interaction. After the adsorption of TA, the IEP reduced to 3.6 (b), which is attributable to the combination of negatively charged TA with the positively charged sites on the cotton modified by HACC.

### 3.3. Adsorption Kinetics Study and Time Saving Estimation

**Figure 4:**
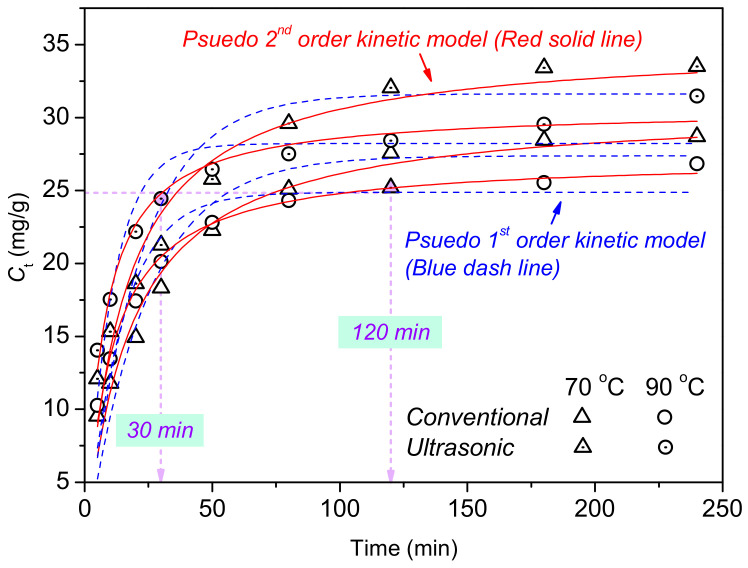
Adsorption kinetics of TA for cationised cotton.

To illustrate the role of ultrasound during the co-depositing, the adsorption rates of TA on cationised cotton under ultrasound and in a waterbath were investigated. *Pseudo 1^st^* and *2^nd^* order kinetic models were used to study the adsorption behaviour of TA on cationised cotton at 70 and 90 °C. The models’ fitness was determined using normalised deviations (*ND*). The kinetic constant (*k*), initial adsorption rate (*hi*), and the half-adsorption time (*t*_1/2_) were calculated from nonlinear fitting to the kinetic models. More details about the calculations are presented in our previous study [9]. The nonlinear fitting curves and the corresponding results are displayed in Figure 4 and Table 3, respectively.

The adsorption of the TA onto the cationised cotton was fast for the first 30 min. An adsorption equilibrium was almost reached after 120 min. As Figure 4 illustrates, the non-linear fitting curves for the *Pseudo* *2^nd^* order kinetic model were closer to the experimental data, compared to those fitted to the *Pseudo* *1^st^* order kinetic model. Due to the higher *R^2^* and smaller *NDs* shown in Table 3, the *Pseudo 2^nd^* order kinetic model was a more suitable model than the *Pseudo* *1^st^* order kinetic model, for investigating the adsorbing behaviour of TA to cationised cotton.

In general, the whole adsorption process was a rate-controlling type which indicated the chemisorption mechanism [29]. As the temperature rose from 70 °C to 90 °C, the *t*_1/2_ decreased dramatically, whilst *k*_2_ and *hi* increased. There were three main reasons for this result: firstly, cotton fibre swells at high temperature, leaving larger pores on fibres for easier adsorption of molecules; secondly, the transfer speed of the TA towards the fibre surface was accelerated; thirdly, the diffusion of the TA to the fibre interior was enhanced. Figure 4 also reveals that the mass of the TA on the cationised cotton at 30 min under ultrasound was close to that at 120 min in the waterbath approach. Thus, around 90 min processing time was saved. At the same temperature, the cloth treated with ultrasound had greater *k*_2_ and *h_i_*, with a shorter *t*_1/2_. Based on the variation of the *k* value, the adsorption efficiency was upgraded by ultrasound by 5.3% (70 °C) and 27% (90 °C) with a corresponding half-adsorption time (*t*_1/2_) decrease of 16.9% (70 °C) and 29.9% (90 °C). The increased adsorption efficiency by ultrasound stemmed from the ultrasonic cavitation: specifically, high-velocity jetting liquid induced by ultrasound generated turbulence and mixing at the fibre-treatment solution interface. The cavitation effect of ultrasound also enabled the aggregated TA molecules, and promoted their diffusion to the core of the fibre [30]. Overall, the ultrasound technology enabled a more efficient approach at lower temperatures than does the conventional procedure (usually carried out at 98 °C for 60 min) [31], which should finally reduce energy and time consumption during textile manufacturing.

### 3.4. CCD Analysis

Based on mathematical modelling, an equation representing the relationship between the treatment conditions and the UPF values (used as an indicator of functional property) of the cotton fabric was created to understand the main factors and their interactions. Response surfaces were used to analyse the results from several experiments, which was more efficient than a single factor experiment [32]. Three variables—TA concentration (1.3~14.7 g/L), pH value (0.7~7.4), and temperature (26.4~93.6 °C)—are indicated as *A*, *B* and *C*, respectively, in Table 1, and a quadratic equation was created as follows:

UPF = υ_0_ + υ_1_*A + υ_2_*B + υ_3_*C + υ_4_*A*B + υ_5_*A*C + υ_6_*B*C + υ_7_*A
^2^+ υ_8_*B
^2^+ υ_9_*C
^2^

where *A*, *B*, and *C* are variables; υ_0_ is the constant; υ_1_, υ_2_, and υ_3_ are the coefficients of linear terms; υ_4,_ υ_5_, and υ_6_ are the coefficients of interactive terms; and υ_7_, υ_8_, and υ_9_ are the coefficients of quadratic terms. The final equation obtained from the modelling is described as follows:

UPF = −108 + 11.03*Conc. − 5.03*pH + 3.69*Temp. − 1.832*Conc.*pH − 0.082*Conc.*Temp. − 0.059*pH*Temp. + 1.201*Conc.*Conc. + 1.530*pH*pH − 0.016*Temp.*Temp.(3)

Except for the interactive coefficient υ_6_ for pH and temperature, the linear coefficients (υ_1_, υ_2_, and υ_3_), the two-way interactive coefficients (υ_4_ and υ_5_), and the square term coefficients (υ_7_, υ_8_, and υ_9_) had a quite small *p*-value (<0.001), indicating their significance in determining the UPF of the cotton fabric. The *p*-value of ‘lack-of-fit’ (0.24) was substantially greater than the threshold (0.05), indicating that ‘lack-of-fit’ was insignificant. The anticipated R-Sq (99.71%) was extremely close to the adjusted R-Sq (99.12 %), indicating that the expected and experimental values were quite consistent. The strong R^2^ (99.85%) suggests that the established model was perfectly predictable.

**Figure 5:**
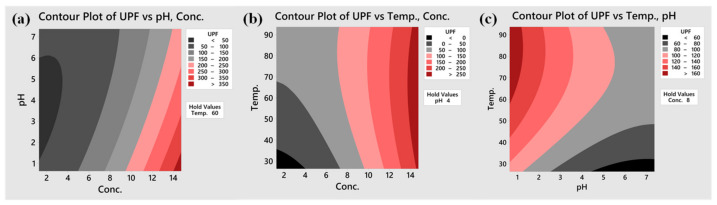
Contour plots for UPF as function of dual factors: (**a**) TA concentration vs. pH, (**b**) TA concentration vs. temperature, and (**c**) pH vs. temperature.

Based on the quadratic model, two-dimensional contour plots describing the impact of factors on the UPF of the cotton fabric were obtained, and are shown in Figure 5. A dark red zone indicating high UPF is located at the bottom right corner, signifying that increased TA concentrations and decreased pH values achieved higher UPF (Figure 5a). This phenomenon was due to the intensified de-ionising degree of the hydroxyls on the cellulose at lower pH, which reduced the repellence of the TA, which was consistent with the ζ-potential variation of the cotton along with the pH change (Figure 3). In addition, temperature rise led to fibre expansion, and accelerated the movement of the molecules in the solution or on the fibres. As depicted in Figure 5b, an interesting phenomenon occurred, whereby increasing the temperature at lower TA concentration achieved a higher UPF value. At higher TA concentration, the highest UPF was obtained at medium temperature. This was due to the fact that the adsorption of the TA to the cotton fibre surface followed by transference to the interior, was more dependable with the expansion of the cotton at low TA concentration. However, when using high TA concentration, the TA molecules at high temperature were prone to be hydrolysed, compromising the UV-proof property. Decreasing the pH value and increasing the temperature to a certain extent enabled higher UPF (Figure 5c). Overall, better UV-shielding performance was achieved using higher TA concentration but at medium temperature.

**Figure 6:**
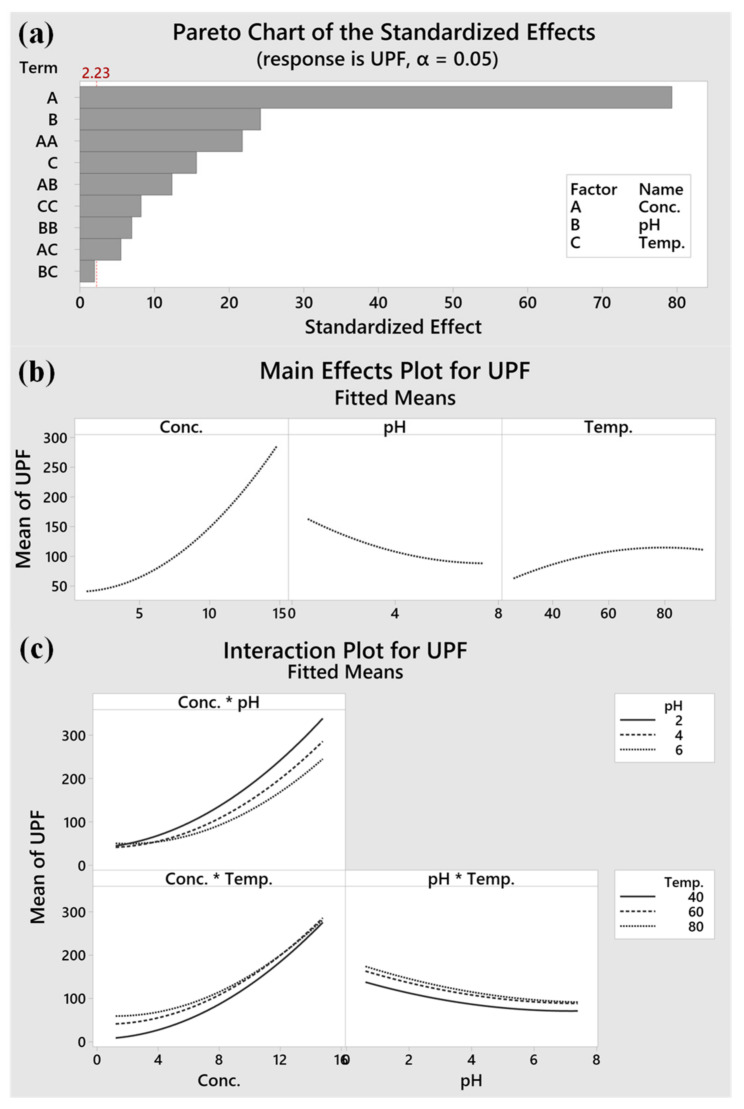
(**a**) Pareto chart, (**b**) Main effects, and (**c**) Interaction plots.

In this section, the significance of the variables and their interactions are explored, based on mathematical modelling. A Pareto chart is utilised to describe the absolute value of the impacts. According to the Pareto chart in Figure 6a, and the main impact plots in Figure 6b, TA concentration (A) was the most noticeable variable, followed by pH (B) and temperature (C). There was no intersection point in the curves of the pH*Temp (Figure 6c), indicating their negligible interactions, which was reconfirmed by the large *p*-value (0.103) in Table 4. Finally, a calculated maximum UPF value of 380 could be achieved at an optimal condition, as follows: TA concentration of 14.7 g/L, pH at 0.7, and temperature at 69.9 °C.

### 3.5. Characterisations

#### 3.5.1. Colour Features

**Figure 7:**
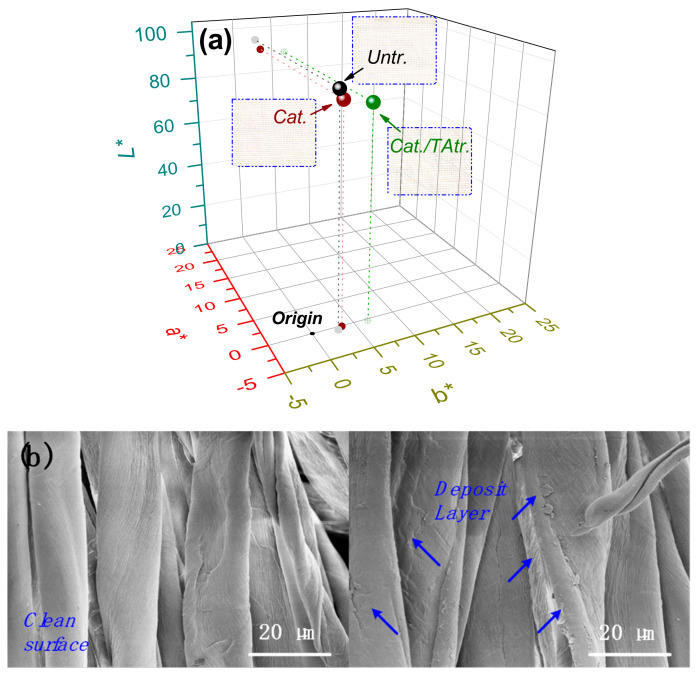
Colour features of Untr., Cat., and Cat./TAtr. cotton fabric: (**a**) L*a*b* values in 3D colour space, and (**b**) SEM images of Untr. (**left**) and Cat./TAtr. (**right**) cotton.

The colouristic coordinates of the cotton fabrics in L*a*b* three-dimensional colour space, with photo inserts, are shown in Figure 7a. In general, the coordinates of the cationised and of the cationised/TA-treated cotton fabrics were located closely to that of the untreated cotton fabric and, correspondingly, their *K/S* values were very low (<1.5), which indicated that negligible colour change occurred on the cotton after treatment. This phenomenon benefited from the colourless natures of HACC and TA, confirmed in their UV–Vis adsorption spectra in Figure 2, and was in alignment with our initial expectation. The SEM images in Figure 7b display a thin layer co-deposition of HACC and TA on the surface of the cotton.

#### 3.5.2. Proposed Interactive Scheme

**Figure 8:**
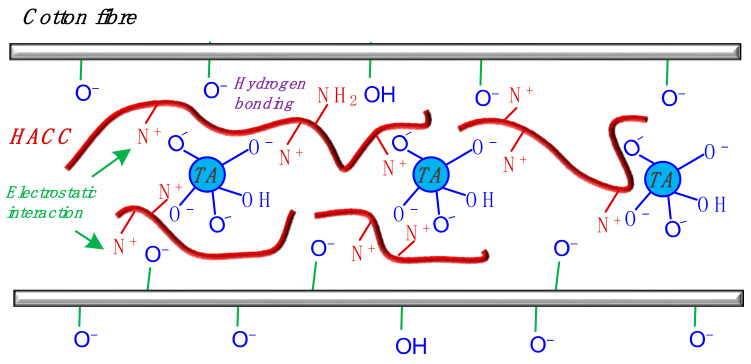
Proposed triple interactive scheme for HACC, TA, and cotton fibres.

Based on the analyses above, a scheme describing the interactions among TA, HACC, and cotton is proposed in Figure 8. Briefly, HACC creates cationic sites (NH_3_^+^) on cotton, through electrostatic attraction, to further bind with TA molecules. Therefore, a three-dimensional physical interlocking structure is generated, which is strong enough to prevent the TA from washing or rubbing out of the fabric during usage.

#### 3.5.3. Antibacterial Activity

**Figure 9:**
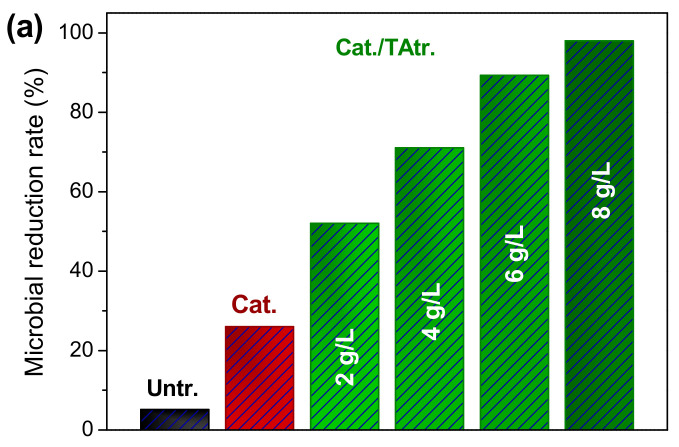

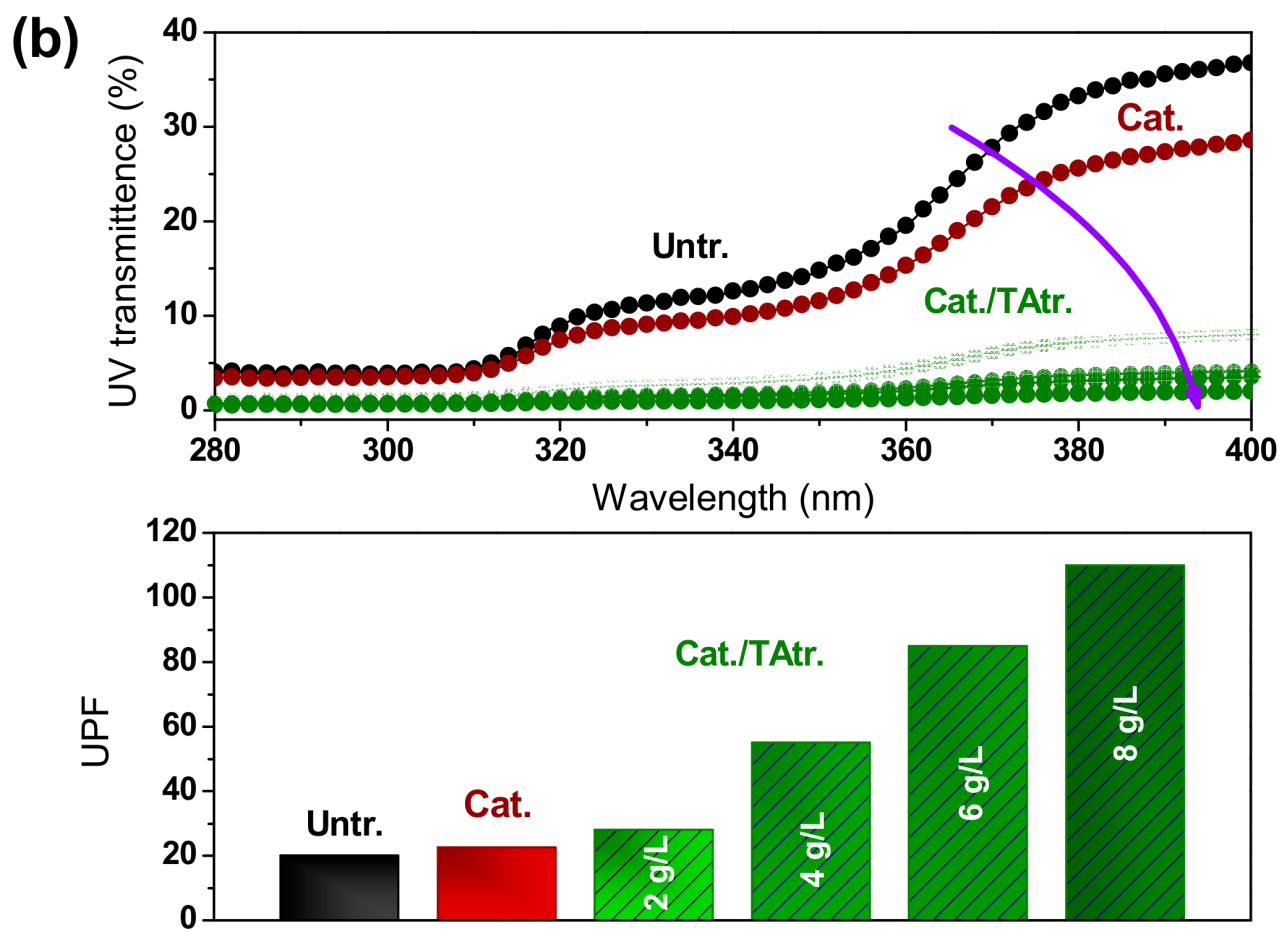
(**a**) Antibacterial and (**b**) UV-protective properties of Untr., Cat., and Cat./TAtr. fabrics.

As described in Figure 9a, untreated cotton fabric showed quite weak bacterial resistance. The cotton treated with HACC disinfected merely 26% of *E. coli* after 24 h contact. Two g/L of TA enhanced the antibacterial activity of the cationised cotton by over 50%. When using the 8 g/L of TA, the antibacterial rate was further promoted by up to 96%. After five washing cycles, the antibacterial activity of the cationised/TA-treated cotton fabric decreased, but was still higher than 80%. The antibacterial mechanism of the TA was related to the disordering of the bacterial membranes, interacting with the extracellular enzymes, taking away necessary substances for bacterial survival, etc.

Further to the theoretical adsorption study in the Section 3.4. CCD Analysis, this section focuses on an in-depth study of the UV-shielding capability of TA-modified cotton fabric from a practical perspective (Figure 9b). TA, due to its strong adsorption within the UV region, is an ideal candidate for UV-proof finishing of textiles (Figure 2). Untreated cotton fabric has high UV transmittance under strong sunlight exposure, which may induce skin carcinogenesis [33]. HACC can barely impart cotton fabric with desirable UV-proof function. The cationised/TA-treated cotton reduces the UV radiation significantly, thus confirming the major contribution of TA to the UV-protective property of cotton fabric. The ultraviolet protection factor (UPF) of cotton fabric is greatly improved, and further increases at higher TA concentration. Therefore, the superior UPF performance demonstrates that cationised/TA-treated fabric can be safely used as protective clothing against UV radiation for outdoor activities.

## 4. Conclusions

This study introduces an efficient fabricating strategy for multifunctional cotton fabric, using TA and HACC, and ultrasonic technology. The results confirm that HACC enhances the affinity of TA to cotton by creating additional cationic binding sites. Negligible colour change was observed after the co-deposition of HACC and TA on cotton. In the adsorption study, the attachment of the TA to the cotton mainly relied on the electrostatic interaction between the HACC and the TA. There was around 5.3% and 27% improvement of adsorption efficiency under ultrasound at 70 °C and 90 °C, respectively. Correspondingly, the half-adsorption time (*t*_1/2_) was shortened by 16.9% (70 °C) and 29.9% (90 °C). According to the CCD experiment, factors influencing UPF were in the following order of significance: concentration > pH > temperature, and ultimately a theoretical maximum UPF value of 380 could be realised on cotton fabric. TA (8 g/L) endows cotton with 98% bacterial reduction and an excellent UV-protective property.

## Figures and Tables

**Table 1:** Variables and experimental design levels.

Variables	Levels				
	−α	−1	0	1	α
X_1_: Conc. (g/L)	1.3	4	8	12	14.7
X_2_: pH	0.7	2	4	6	7.4
X_3_: Temp. (°C)	26.4	40	60	80	93.6

**Table 2:** Composition of various runs; predicted and actual responses.

Run	Conc. (g/L)	pH	Temp. (°C)	UPF	
				Predicted	Actual
1	4	2	40	38.6	40.6
2	12	2	40	225.0	227.1
3	4	6	40	28.6	32.2
4	12	6	40	156.5	156.4
5	4	2	80	84.3	84.5
6	12	2	80	244.6	241.1
7	4	6	80	65.0	63.0
8	12	6	80	166.7	164.7
9	1.3	4	60	41.0	38.8
10	14.7	4	60	283.3	285.4
11	8	0.7	60	162.1	161.7
12	8	7.4	60	88.2	88.5
13	8	4	26.4	64.5	60.0
14	8	4	93.6	111.6	116.0
15	8	4	60	107.8	107.0
16	8	4	60	107.8	105.0
17	8	4	60	107.8	112.0
18	8	4	60	107.8	110.0
19	8	4	60	107.8	109.0
20	8	4	60	107.8	104.0

**Table 3:** Kinetic parameters for the adsorption of TA to cationised cotton.

	70 °C		90 °C	
**Ultrasound**	**No**	**Yes**	**No**	**Yes**
*Pseudo 1^st^ order kinetics*				
*k*_1_ (/min)	0.0425	0.0477	0.0701	0.0969
*C*_e_ (mg/g)	27.37	31.62	24.88	28.21
*R* ^2^	0.91	0.85	0.91	0.85
*ND* (%)	5.00	4.95	3.15	2.54
*Pseudo 2^nd^ order kinetics*				
*k*_2_ (g/[mg⋅min])	0.0019	0.0020	0.0037	0.0047
*C*_e_ (mg/g)	30.73	35.12	27.26	30.61
*h*_i_ (mg/[g⋅min])	1.79	2.47	2.75	4.40
*t*_1/2_ (min)	17.13	14.24	9.91	6.95
*R* ^2^	0.97	0.95	0.98	0.97
*ND* (%)	3.01	2.72	1.22	0.98

**Table 4:** ANOVA for response surface quadratic model.

Source	DF	Adj SS	Adj MS	F-Value	*p*-Value
Model	9	89,065.6	9896.2	716.05	0.000
Linear	3	80,117.9	26,706.0	1932.33	0.000
Conc.	1	70,849.3	70,849.3	5126.36	0.000
pH	1	6593.1	6593.1	477.05	0.000
Temp.	1	2675.5	2675.5	193.58	0.000
Square	3	6842.5	2280.8	165.03	0.000
Conc. * Conc.	1	5317.9	5317.9	384.78	0.000
pH * pH	1	540.0	540.0	39.07	0.000
Temp. * Temp.	1	706.3	706.3	51.10	0.000
2-Way Interaction	3	2105.3	701.8	50.78	0.000
Conc. * pH	1	1719.0	1719.0	124.38	0.000
Conc. * Temp.	1	341.7	341.7	24.72	0.001
pH * Temp.	1	44.5	44.5	3.22	0.103
Error	10	138.2	13.8		
Lack-of-Fit	5	91.4	18.3	1.95	0.240
Pure Error	5	46.8	9.4		
Total	19	89,203.8			
Model Summary					
		S	R-sq	R-sq (adj)	R-sq (pred)
		3.7176	99.85%	99.71%	99.13%

## Data Availability

All data that support the findings of this study are available from the corresponding authors upon reasonable request.
